# Biomass‐Derived Multilayer‐Structured Microparticles for Accelerated Hemostasis and Bone Repair

**DOI:** 10.1002/advs.202002243

**Published:** 2020-10-04

**Authors:** Jia‐Ying Liu, Yang Hu, Long Li, Chao Wang, Jia Wang, Yang Li, Dafu Chen, Xiaokang Ding, Chuanan Shen, Fu‐Jian Xu

**Affiliations:** ^1^ State Key Laboratory of Chemical Resource Engineering Key Laboratory of Biomedical Materials of Natural Macromolecules (Beijing University of Chemical Technology, Ministry of Education) Beijing Laboratory of Biomedical Materials Beijing University of Chemical Technology Beijing 100029 China; ^2^ Laboratory of Bone Tissue Engineering Beijing Laboratory of Biomedical Materials Beijing Research Institute of Traumatology and Orthopaedics Beijing Jishuitan Hospital Beijing 100035 China; ^3^ Department of Burn & Plastic Surgery The First Affiliated Hospital of General Hospital of PLA Beijing 100048 China

**Keywords:** bleeding, bone defects, plant polyphenols, starch, tissue repair

## Abstract

It is very desirable to develop advanced sustainable biomedical materials with superior biosafety and bioactivity for clinical applications. Herein, biomass‐derived multilayer‐structured absorbable microparticles (MQ*_x_*T*_y_*) composed of starches and plant polyphenols are readily constructed for the safe and effective treatment of bone defects with intractable bleeding by coating multiple layers of quaternized starch (Q^+^) and tannic acid onto microporous starch microparticles via facile layer‐by‐layer assembly. MQ*_x_*T*_y_* microparticles exhibit efficient degradability, low cytotoxicity, and good blood compatibility. Among various MQ*_x_*T*_y_* microparticles with distinct Q^+^/T^−^ double layers, MQ_2_T_2_ with outmost polyphenol layer possess the unique properties of platelet adhesion/activation and red blood cell aggregation, resulting in the best hemostatic performance. In a mouse cancellous‐bone‐defect model, MQ_2_T_2_ exhibits the favorable hemostatic effect, low inflammation/immune responses, high biodegradability, and promoted bone repair. A proof‐of‐concept study of beagles further confirms the good performance of MQ_2_T_2_ in controlling intractable bleeding of bone defects. The present work demonstrates that such biomass‐based multilayer‐structured microparticles are very promising biomedical materials for clinical use.

## Graphical abstract

Biomass‐derived multilayer‐structured hemostatic microparticles (MQ_2_T_2_) composed of starches and plant polyphenols are constructed to integrate the advantages of cationized amylopectin (with high viscosity), polyphenol (with unique properties in accelerating hemostasis), and multilayer structures (with favorable biodegradability), which can achieve effective (accelerated hemostasis and promoted bone repair) treatments of bone defects with intractable bleeding in mouse and beagle models.

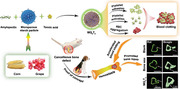

## 1 Introduction

More and more biomedical materials have been explored as major supplements to surgical techniques for intractable bleeding conditions.^[^ ^1^, ^2^, ^3^, ^4^ ^]^ For instance, bone wax, consisting of paraffin and esterified fatty acids, is widely used in almost all fields of surgery due to its low cost and ease of use.^[^ ^5^ ^]^ However, bone wax has been associated with inflammation, granuloma formation, hindered osteogenesis and even bone wax migration.^[^ ^6^, ^7^ ^]^ Possible substitutes of bone wax have been constructed from petroleum‐based polymers and inorganic materials,^[^ ^8^, ^9^ ^]^ which still possess some inherent shortcomings including slow or incomplete degradation and poor effects on anti‐inflammation/wound healing. Moreover, biomass‐based materials had been particularly appealing for an economical and sustainable society of mankind.^[^ ^10^, ^11^ ^]^ It is very desirable to develop promising advanced biomass‐based biomedical materials with superior biosafety and bioactivity for clinical applications.

A variety of polysaccharides have been produced from renewable biomass and appealing in exploring biomaterials, due to their high biocompatibility and unique bioactivity in human body.^[^ ^12^, ^13^ ^]^ Among them, starch is recently recognized as an ideal polysaccharide to construct biomaterials due to its excellent biodegradability and biosafety from plant origin.^[^ ^14^, ^15^, ^16^, ^17^ ^]^ A class of starch‐based materials were designed for hemostatic use, including cross‐linked starch microparticles and calcium ion‐loaded starch particles.^[^ ^16^, ^17^ ^]^ However, they were limited by the application for real‐time bleeding due to their simple structures. Apart from polysaccharides, naturally occurring polyphenols as the secondary metabolites of plants have also received increasing attentions in biomedical field, owing to their unique bioactivities such as anti‐inflammatory, antimicrobial and antioxidant activities.^[^ ^18^ ^]^ As a widely used polyphenol, tannic acid (T^−^) has been applied in facile surface modification of biomaterials via T^−^/metal ions complex or T^−^/cationic species layer‐by‐layer assembly.^[^ ^19^, ^20^ ^]^ T^−^‐based biomaterials were reported with good biocompatibility/biodegradability, high affinity for various proteins/cells and promoted wound healing.^[^ ^21^, ^22^, ^23^ ^]^ Thus, it is possible to develop multifunctional biomedical materials based on biomass‐derived starch and T^−^ to realize effective treatments of intractable bleeding.

**Figure 1:**
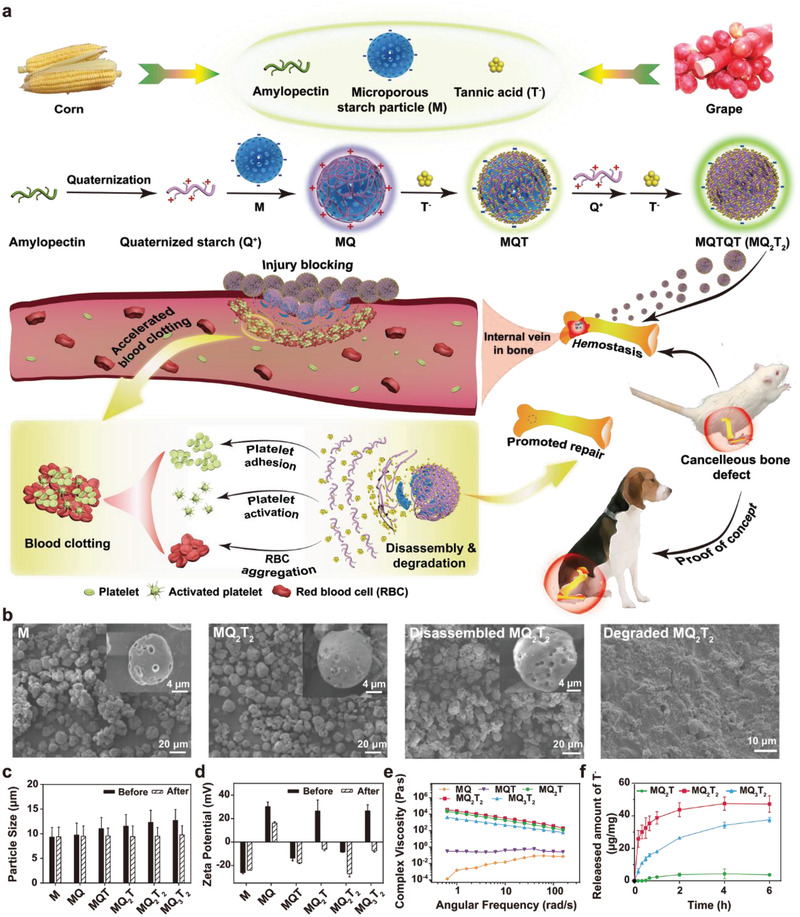
a) Schematic diagram illustrating the synthetic route of biomass‐derived multilayer‐structured hemostatic microparticles and their applications in cancellous bone defect repair. b) SEM images of M, MQ_2_T_2_, disassembled MQ_2_T_2_ incubated with PBS at 37 °C for 6 h (magnified images in the inserts) and degraded MQ_2_T_2_ treated with glucoamylase/amylase for 16 h. c) Particle size (*n* ≥ 50) and d) zeta potential (*n* ≥ 3) of M and MQ*_x_*T*_y_* microparticles before and after incubation with PBS. e) Complex viscosities of M and MQ*_x_*T*_y_* microparticles in water. f) Released amount of T^−^ of MQ_2_T, MQ_2_T_2_, and MQ_3_T_2_ microparticles in PBS (*n* = 4).

Bone defect is one kind of frequently occurring disease following trauma, inflammatory/infection processes and tumor resection.^[^ ^24^, ^25^ ^]^ Due to the abundant blood vessels and systolic blood pressure in cancellous bones, bone‐defect‐induced bleeding is clinically recognized as intractable bleeding due to its sustained bleeding behavior before long‐term bone repair.^[^ ^8^, ^26^ ^]^ Herein, biomass‐derived multilayer‐structured hemostatic microparticles (MQ*_x_*T*_y_*) composed of starches and plant polyphenols were flexibly constructed by layer‐by‐layer assembly based on negatively charged microporous starch particle (M) cores and multiple quaternized starch (Q^+^)/T^−^ layers (**Figure** **1**a). Such absorbable microparticles were proposed as advanced biomedical materials to integrate the advantages of M, Q^+^, and T^−^ and achieve effective treatments of bone defects with intractable bleeding. Q^+^ layer was prepared by the quaternization of amylopectin to endow MQ*_x_*T*_y_* microparticles with high viscosity. The optimal hemostatic property was screened from MQ*_x_*T*_y_* microparticles with distinct Q^+^/T^−^ double layers. Furthermore, the combination of M and Q^+^/T^−^ layers from plants would enable MQ*_x_*T*_y_* microparticles with favorable biocompatibility/biodegradability/bioactivities of starch and polyphenol, which also probably benefits the controlling of sustained bleeding of bone defects and subsequently accelerates bone repair in mouse and beagle models.

## 2 Results and Discussion

**Figure 2:**
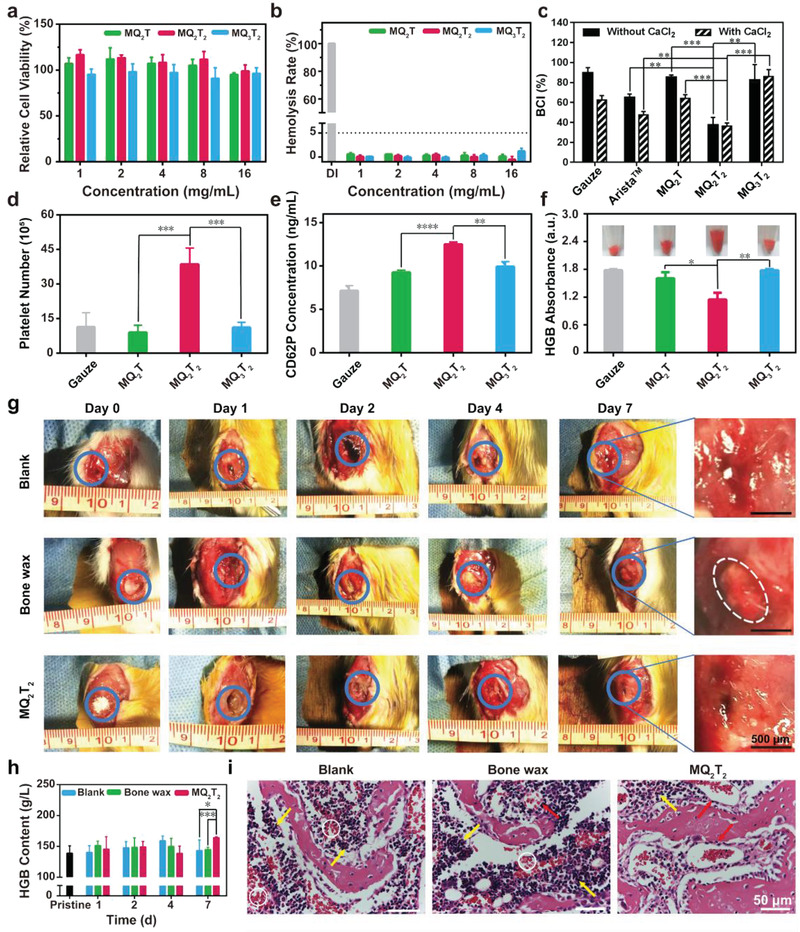
a) Cell viabilities and b) hemolysis rates of the MQ_2_T, MQ_2_T_2_, and MQ_3_T_2_ microparticles at various concentrations (*n* = 3–4). c) BCI index (*n* = 3), d) adherent platelet number (*n* = 4) and e) CD62P content of whole blood (*n* = 3) treated with the gauze, MQ_2_T, MQ_2_T_2_ and MQ_3_T_2_ groups. f) HGB content of adherent RBCs in the gauze, MQ_2_T, MQ_2_T_2_, and MQ_3_T_2_ groups after incubation with diluted whole blood (photographs are inserted) (*n* = 3). g) Representative photographs of bone apertures (blue circle indicates bone defect area and white circle indicates residual bone wax) and h) HGB contents (*n* = 3–7) of whole blood samples in the Blank, Bone wax and MQ_2_T_2_ groups during Day 0–7. i) H&E staining analysis of the defected bone samples in the Blank, Bone wax and MQ_2_T_2_ groups at Day 4 (white circle indicates bleeding sites, yellow arrow indicates inflammation‐associated cells and red arrow indicates repaired blood vessels). Data are presented as the mean ± SD. *p < 0.05, **p < 0.01, ***p < 0.001, ****p < 0.0001 among the marked groups using multiple *t* tests.

The detailed synthetic strategy of MQ*_x_*T*_y_* microparticles was illustrated in Figure 1a. Q^+^ with the substitution degree of quaternary ammonium of 0.33 was obtained by reacting amylopectin with glycidyl trimethyl ammonium chloride (GTA) and characterized by NMR (Figure S1, Supporting Information). M with one Q^+^ layer (MQ) was then prepared by the electrostatic assembly of Q^+^ and negatively charged M in deionized (DI) water. Finally, MQ*_x_*T*_y_* microparticles with multiple Q^+^/T^−^ layers (MQT, MQ_2_T, MQ_2_T_2_ and MQ_3_T_2_) were prepared by layer‐by‐layer assembly of Q^+^ and T^−^ in sequence. SEM images demonstrated the feature of microporous spherical morphology of M and MQ*_x_*T*_y_* microparticles (**Figure** **2**b; Figure S2a, Supporting Information). Obviously, the pore number of MQ*_x_*T*_y_* microparticles decreased with the increase of Q^+^/T^−^ layers. Compared with M, the average particle sizes of MQ*_x_*T*_y_* microparticles became bigger with the increase of Q^+^/T^−^ layers (Figure 1c; Figure S2b, Supporting Information). Meanwhile, the zeta potentials of MQ*_x_*T*_y_* microparticles were alternately positive or negative due to the variation of outmost charged layers (Figure 1d). All the results indicated that MQ*_x_*T*_y_* microparticles were well prepared via lay‐by‐layer assembly. It is worth mentioning that MQ*_x_*T*_y_* microparticles with more Q^+^/T^−^ layers (i.e., MQ_3_T_3_) were found to be unstable, probably due to the limited electrostatic interactions among the M, low‐substitution‐degree Q^+^ and T^−^ species. In addition, based on the typical ^13^C NMR spectra of M, Q^+^, T^−^ and MQ_2_T_2_ microparticles, it was confirmed that MQ_2_T_2_ was composed of M, Q^+^, and T^−^ (Figure S3, Supporting Information).

Rheological analysis of MQ*_x_*T*_y_* microparticles in water was performed to compare their dispersion states and viscosities in liquid. MQ and MQT exhibited similar storage module (*G*′) and loss module (*G*′′) (Figure S4, Supporting Information), indicating a solid‐like structure without floating. On the contrary, the G′ values of MQ_2_T, MQ_2_T_2_, and MQ_3_T_2_ microparticles were obviously greater than the corresponding *G*′′ counterparts. As shown in Figure 1e, the complex viscosities of MQ_2_T, MQ_2_T_2_, and MQ_3_T_2_ microparticles were obviously higher than those of MQ and MQT microparticles, which decreased with the increase of angular frequency. These high complex viscosity and shear thinning phenomenon and high complex viscosity further confirmed that MQ_2_T, MQ_2_T_2_, and MQ_3_T_2_ microparticles were elastic, high‐viscosity and of good solid‐like mechanical response. It can be inferred that an adequate amount/layer number of Q^+^ layer is crucial for the high‐viscosity and gel‐like state of MQ*_x_*T*_y_* microparticles. Considering the powder form (unlike commonly hemostatic membrane or sponge) of MQ*_x_*T*_y_* microparticles, MQ_2_T, MQ_2_T_2_, and MQ_3_T_2_ microparticles with gel‐like state would produce the effective mixture with blood as a whole, which is favorable for the function of their potential hemostatic effect. Thus, MQ_2_T, MQ_2_T_2_, and MQ_3_T_2_ microparticles were subsequently evaluated as as potential hemostatic particles.

Due to the electrostatic assembly, MQ*_x_*T*_y_* microparticles was proposed to disassemble and release Q^+^/T^−^ layers in physiological conditions. After incubation with PBS, all three MQ*_x_*T*_y_* microparticles exhibited similar microporous morphologies to that of pristine M (Figure 1b; Figure S5a, Supporting Information). The particle sizes of disassembled MQ*_x_*T*_y_* microparticles were similar to that of pristine M after the treatment with PBS (Figure 1c; Figure S5b, Supporting Information), while the zeta potentials of MQ_2_T and MQ_3_T_2_ decreased to be below zero (Figure 1d). In addition, Figure 1f displayed the accumulative T^−^ release of MQ_2_T, MQ_2_T_2_, and MQ_3_T_2_ microparticles after incubation with PBS, of which the different T^−^ release rates are consistent with their distinct construction of T^−^ layers. All the above results demonstrate that the Q^+^ and T^−^ layers of MQ*_x_*T*_y_* microparticles can be successfully disassembled. Enzymolysis assay was further performed by the incubation of MQ_2_T_2_ with glucoamylase and *α*‐amylase.^[^ ^17^ ^]^ The hydrolyzed MQ_2_T_2_ microparticles were observed under SEM at different hydrolysis periods (Figure 1b; Figure S6, Supporting Information). MQ_2_T_2_ microparticles were totally broken at the short period of 16 h, demonstrating the good degradability of MQ*_x_*T*_y_* microparticles.

The in vitro biocompatibility of MQ*_x_*T*_y_* microparticles was confirmed by cytocompatibility and blood compatibility assays. As shown in Figure 2a, the cell viabilities of MQ_2_T, MQ_2_T_2_, and MQ_3_T_2_ microparticles were evaluated with typical fibroblast (L929) cells. MQ_2_T, MQ_2_T_2_, and MQ_3_T_2_ microparticles exhibited high cell viabilities (>90%) at the high concentration of 16 mg mL^−1^, demonstrating the excellent cytocompatibility of MQ*_x_*T*_y_* microparticles. Normally, low hemolysis ratio (<5%) of red blood cells (RBC) has been recognized as qualified blood compatibility of biomaterials.^[^ ^27^ ^]^ All MQ_2_T, MQ_2_T_2_, and MQ_3_T_2_ microparticles showed low hemolysis ratios of at most 2% at various concentrations (Figure 2b), confirming their good blood compatibility.

The in vitro hemostatic property of MQ_2_T, MQ_2_T_2_, and MQ_3_T_2_ microparticles was assessed by blood‐clotting index (BCI) assay. Herein, medical gauze and commercial starch‐based absorbable hemostat (Arista) were used as the control groups for in vitro hemostatic assay. Bone wax was not selected as control because its hemostatic effect arises from physical sealing of capillary injury.^[^ ^5^ ^]^ MQ*_x_*T*_y_* microparticles and medical gauze (as the control group) were incubated with the same amount of citrated whole blood (extracted from healthy SD rats) or recalcified whole blood (citrated whole blood mixed with 10% 0.2 m CaCl_2_ solution), to measure BCI_(without CaCl2)_ or BCI_(with CaCl2)_, respectively.^[^ ^28^, ^29^ ^]^ A low BCI value indicates high in vitro hemostatic property. Recalcified whole blood starts to clot immediately after the addition of CaCl_2,_ while citrated whole blood keeps unclotted due to the lack of Ca^2+^/inhibition of coagulation system. Thus, BCI_(with CaCl2) and_ BCI_(without CaCl2)_ partially reflect the corrsponding hemostatic property of materials dependent/independent of coagulation system, the latter of which is important to treat serious bleeding (i.e., massive hemorrhage and chronic bleeding).^[^ ^29^ ^]^ As shown in Figure 2c, medical gauze and Arista exhibited a high BCI_(without CaCl2)_ and a low BCI_(with CaCl2)_, indicating the moderate hemostatic property in regular bleeding but low hemostatic property in serious bleeding, which is consistent with their clinical use for minor hemorrhage. Interestingly, MQ_2_T_2_ group showed similar BCI values regardless of CaCl_2_, indicating the hemostatic property of MQ_2_T_2_ microparticles was independent of coagulation system. More importantly, the BCI values of MQ_2_T_2_ group were significantly lower than those of Arista and MQ_2_T/MQ_3_T_2_ groups, demonstrating the superior hemostatic property of MQ_2_T_2_ among three MQ*_x_*T*_y_* microparticles.

To explore the underlying hemostatic mechanism of different MQ*_x_*T*_y_* microparticles, the platelet adhesion/activation and RBC adhesion/aggregation assays were performed. In comparison with the control gauze with negatively charged surfaces of ∼‐20 mV (due to carboxyl groups), MQ_2_T and MQ_3_T_2_ exhibited the similar number of adhered platelets, indicating the ordinary platelet adhesion ability of Q^+^ layer (Figure 2d). On the other hand, the number of adhered platelets of MQ_2_T_2_ with outmost T^−^ layer was significantly higher than those of MQ_2_T/MQ_3_T_2_, despite of negatively charged surfaces. As shown in Figure 2e, all MQ*_x_*T*_y_* microparticles displayed the higher amount of CD62P protein (a platelet‐activation factor^[^ ^30^ ^]^) than gauze (Figure 2e) after the incubation with platelets, indicating their higher degrees of platelet activation. Moreover, the degree of platelet activation of MQ_2_T_2_ was significantly higher than those of MQ_2_T/MQ_3_T_2_. A modified positive‐adhesion assay was further adopted to evaluate the RBCs aggregation/adhesion property of MQ*_x_*T*_y_* microparticles.^[^ ^31^ ^]^ As shown in Figure 2f, MQ_2_T_2_ showed a significantly lower number of non‐adhesion RBCs (qualified by HGB content) than MQ_2_T/MQ_3_T_2_, indicating its highest RBC aggregation property. Moreover, a high amount of RBCs were directly observed to adhere onto MQ_2_T_2_ particles (Figure 2f; Figure S7, Supporting Information), while only a few RBCs existed in the MQ_2_T/MQ_3_T_2_ groups.

All these results confirmed that MQ_2_T_2_ with outmost T^−^ layer possessed great advantages over MQ_2_T/MQ_3_T_2_ in platelet adhesion/activation and RBC aggregation. Blood proteins were involved in the platelets adhesion/activation and RBCs aggregation assays to simulate/parse the whole blood clotting process. Plasma proteins were reported to compete with RBCs for the interactions with cationic ligands.^[^ ^32^ ^]^ The lower RBC aggregation property of MQ_2_T/MQ_3_T_2_ over MQ_2_T_2_ indicated that due to the strong electrostatic interaction, the positively charged Q^+^ layer would bind faster with plasma proteins than T^−^ layer. In addition, fibrinogen had been recognized as the major plasma protein responsible for platelets adhesion/activation, which requires the mild interaction with material surfaces and the subsequent conformational change.^[^ ^33^ ^]^ Considering the distinct Q^+^/T^−^ double layers of three MQ*_x_*T*_y_* microparticles, MQ_2_T_2_ with outmost T^−^ layer probably produces moderate interaction with plasma proteins and thus facilitates platelets adhesion/activation. It is worth mentioning that all three MQ*_x_*T*_y_* microparticles were confirmed to disassemble and release Q^+^/T^−^ layers in physiological conditions due to the electrostatic assembly (Figure 1e). It can be further inferred that the appropriate mixture of Q^+^/T^−^ layers (i.e., those dissembled from MQ_2_T_2_ with a high proportion of T^−^), not the outmost T^−^ layer alone, would be the key procoagulant component. All the results confirmed the potent in vitro hemostatic property of MQ_2_T_2_, which would contribute to in vivo intractable bleeding treatment.

The in vivo cancellous bone defect with a cylindrical bone aperture (Φ2*3 mm) was built in SD rats to investigate the hemostatic effect of MQ_2_T_2_ on intractable bleeding The rats were randomly assigned to three groups: Blank (without any treatment), Bone wax (aperture filled with bone wax) and MQ_2_T_2_ (aperture filled with MQ_2_T_2_). For the Blank group, bone apertures were observed with blood congestions (indicated by blue circles) at Day 1–4 after operation (Figure 2g), indicating the durative bleeding after the cancellous bone defect. On the contrary, no obvious blood congestion was observed in both Bone wax and MQ_2_T_2_ groups during the 7‐day healing period. Due to the sustained bleeding behavior before long‐term bone repair, it is hard to measure the bleeding time/blood loss in mouse cancellous‐bone‐defect model. The whole blood samples of rats were collected and analyzed after operation, where low HGB level is normally related to post‐operation bleeding or anemia. A significantly higher level of HGB was observed in the MQ_2_T_2_ group than those in the Blank and Bone wax groups at Day 7 (Figure 2h). This result indicated the better capacity of MQ_2_T_2_ to restore/produce HGB, probably due to reduced blood loss during healing process. In addition, the H&E staining photographs were also employed to evaluate the bleeding‐to‐healing process of bone samples (Figure 2i; Figure S8, Supporting Information). At Day 1 and Day 2, a large area of bleeding sites (white circles) and few complete blood vessels (red arrows) were observed in three groups, indicating that the intractable bleeding was continuing. The quantitative analysis revealed that the MQ_2_T_2_ group showed the smaller area of bleeding sites area and more repaired/complete blood vessels at defected areas than the Bone wax group since Day 4 (Figure S9, Supporting Information). All the results demonstrated the good hemostatic property of MQ_2_T_2_ in mouse cancellous‐bone‐defect model.

**Figure 3:**
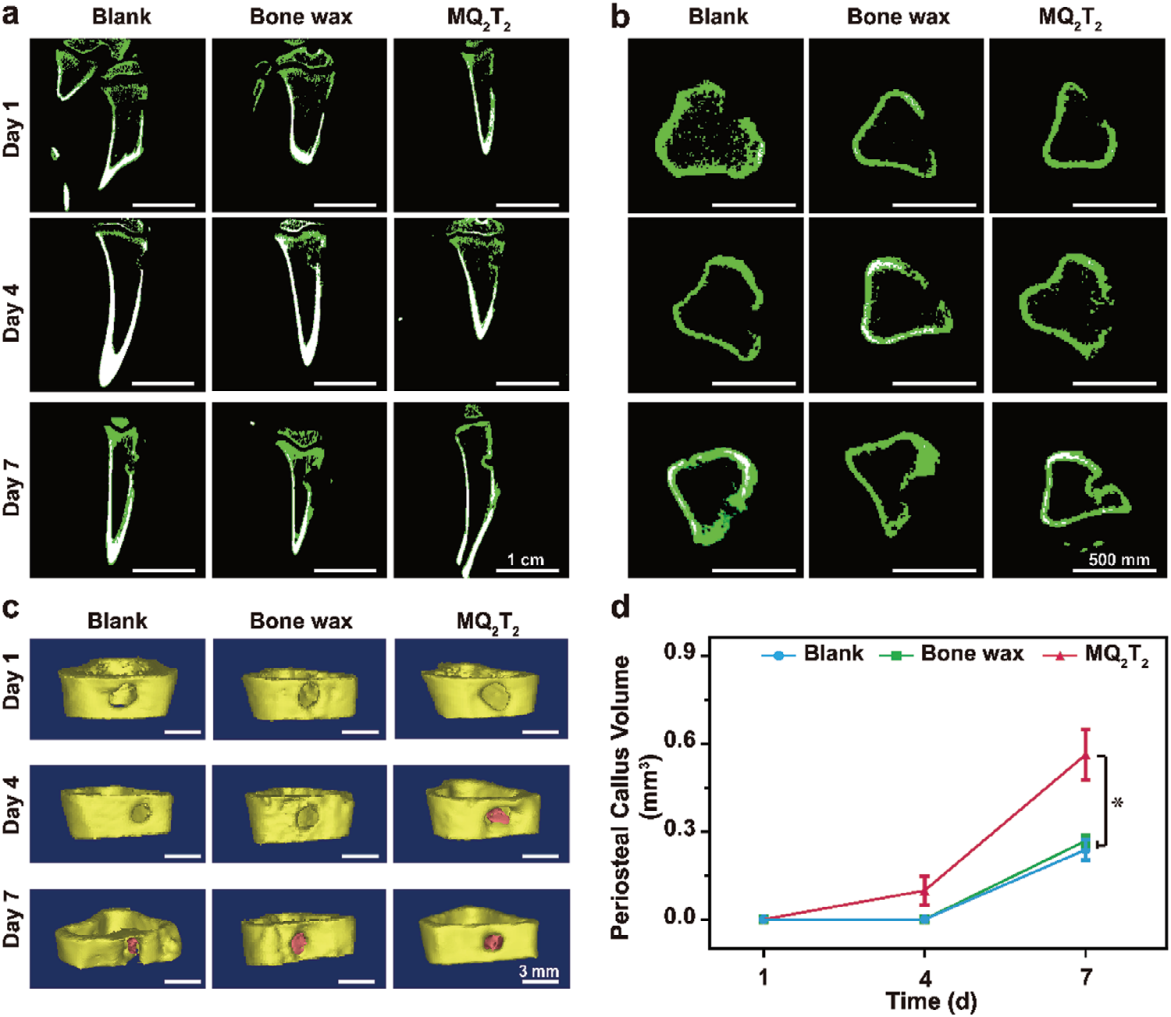
a) Micro‐CT images by a) coronal and b) transverse sections of the defected bone samples in the Blank, Bone wax and MQ_2_T_2_ groups during Day 1–7. c) 3D‐reconstructed images of bone apertures (yellow part indicates original bone and pink part indicates periosteal callus) and d) calculated volumes of periosteal callus in the Blank, Bone wax and MQ_2_T_2_ groups during Day 1–7 (yellow part indicates original bone and pink part indicates periosteal callus) (*n* = 3). Data are presented as the mean ± SD.*p < 0.05 among the marked group using multiple *t* tests.

Considering the sustained bleeding behavior before long‐term bone repair, an ideal hemostatic material for bone defects should also possess a positive effect on bone repair. Micro‐CT was used to assess the periosteal callus formation of rats from three groups after operation. Obvious bone apertures were observed in both three groups at Day 1, confirming the well‐produced bone‐defect model (**Figure** **3**a,b). At Day 4, a small amount of periosteal callus (new bones) on the aperture were only observed in the MQ_2_T_2_ group. As shown in Figure 3c,d, new and original bones of bone samples were differentiated by bone density and 3D‐reconstructed. The MQ_2_T_2_ group exhibited a significantly higher volume of periosteal callus than the Bone wax/Blank groups at Day 7. All the micro‐CT results demonstrated that MQ_2_T_2_ possessed the better effect on bone repair than commercial bone wax.

**Figure 4:**
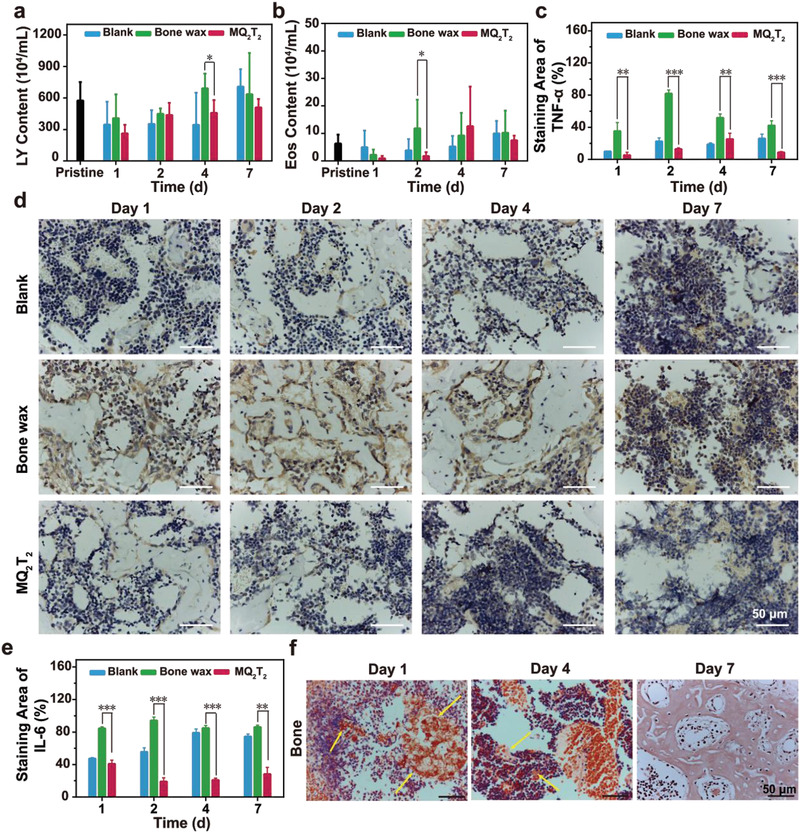
a) LY and b) Eos contents of whole blood samples of defected bone samples in the Blank, Bone wax, and MQ_2_T_2_ groups during Day 1–7 (*n* = 3–7). c) TNF‐*α* staining area (*n* = 3–7), d) immunohistochemical staining analysis (brown part indicates TNF‐*α* expression) and e) IL‐6 staining area (*n* = 3–5) of defected bone samples in the Blank, Bone wax and MQ_2_T_2_ groups during Day 1–7. f) Congo red staining of bone samples in the MQ_2_T_2_ group during Day 1–7, where yellow arrow indicates stained MQ_2_T_2_. Data are presented as the mean ± SD. *p < 0.05, **p < 0.01, ***p < 0.001 among the marked groups using multiple *t* tests.

The biosafety of MQ_2_T_2_ in cancellous‐bone‐defect model was evaluated by inflammation/immune response and biodegradablity assays. At Day 1, a large number of inflammation‐associated cells (indicated by yellow arrow) were observed in the Blank and Bone wax groups, while fewer cells existed in the MQ_2_T_2_ group (Figure S8, Supporting Information). Since Day 4, inflammation‐associated cells were hardly observed in the MQ_2_T_2_ group when the impaired blood vessels were recovered (Figure 2i). For the Bone wax group, there still existed many inflammation‐associated cells around the blood vessels at Day 7 (Figure S8, Supporting Information). The results of histological analysis confirmed that MQ_2_T_2_ exhibited obviously less inflammation‐associated cells than the Bone wax group during healing process. The contents of lymphocyte (LY) and eosinophilic granulocyte (Eos) cells in whole blood samples are also selected as physiological indexes to evaluate the levels of inflammation‐related cells at postoperative phase.^[^ ^34^, ^35^ ^]^ As shown in **Figure** **4**a, the MQ_2_T_2_ group showed only significantly lower LY contents than the Bone wax group at Day 4. Moreover, the Eos contents of MQ_2_T_2_ group were significantly lower than those of the Bone wax group at Day 2 (Figure 4b). The Eos contents of the MQ_2_T_2_ and Bone wax groups were not significantly different at Day 4 or Day 7 (*P* value = 0.51 or 0.28), which is diffent from the histological analysis. Such difference could be attributed to the local treatment of MQ_2_T_2_ at bone apertures. The histological analysis would be more suitable to evaluate the real‐time levels of inflammation‐related cells in bone tissues during healing process.

Tumor necrosis factor‐*α* (TNF‐*α*) and interleukin‐6 (IL‐6) were selected to evaluate the levles of inflammation‐ or immunoregulatory‐associated proteins/cytokines.^[^ ^36^, ^37^ ^]^ TNF‐*α* and IL‐6 expression results were first evaluated by the immunohistochemical analysis of bone sections (Figure 4c–e; Figure S10, Supporting Information). Bone wax group displayed clearly expressed TNF‐*α* and IL‐6 at Day 1–7, while no obvious expressions were observed in those of the Blank and MQ_2_T_2_ groups (Figure 4d; Figure S10, supporting information). The quantitative analysis of bone sections revealed that the percentages of TNF‐*α*‐positive (or IL‐6‐positive) area in the Bone wax group were significantly higher than those in the Blank and MQ_2_T_2_ groups at each time points (Figure 4c,e). In addition, these immunohistochemical results were consistent with the whole blood analysis of TNF‐*α*/IL‐6 (Figure S11, Supporting Information), demonstrating the low expression of inflammation or immunoregulatory‐associated cytokines in the MQ_2_T_2_ group. All the above results confirmed that the MQ_2_T_2_ group exhibited obviously lower inflammation/immune responses than the Bone wax group during the healing process. Such good bioactivity of MQ_2_T_2_ could be attributed to the efficient disassembly/degradation process of MQ_2_T_2_ in physiological condition (Figure 1) and the anti‐inflammatory/antioxidant activities of released T^−^ layer.^[^ ^18^ ^]^

Direct visualization of degradable process of MQ_2_T_2_ microparticles in the bone and surrounding muscles was performed using Congo Red staining. MQ_2_T_2_ microparticles were stained to be red in the bone and muscle sections due to their polysaccharide (Q^+^ and M) components (Figure 4f; Figure S12, Supporting Information). A large number of red‐stained dots were obviously observed at Day 1, while they gradually decreased during Day 2–7. Particularly, all red‐stained starch disappeared at Day 7 from the tissues. These results clearly demonstrated that MQ_2_T_2_ can be efficiently degraded in the bone and muscle tissues.

The mouse cancellous‐bone‐defect model showed that the MQ_2_T_2_ treatment exhibited not only better hemostatic performance than bone wax, but also significantly promoted bone repair. Due to its physical sealing effect on capillary injury, bone wax is applied in bone defect clotting to achieve hemostatic performance in bone‐defect‐induced bleeding.^[^ ^5^ ^]^ However, bone wax was reported with several drawbacks that would hinder bone repair process, such as the lack of biodegradability and risk of inflammation.^[^ ^6^, ^7^ ^]^ On the contrary, the hemostatic property of MQ_2_T_2_ relied on the effective aggregation of blood components including platelets and RBCs, which could contribute to callus formation.^[^ ^24^ ^]^ Moreover, dysregulated inflammation response or prolonged inflammation phase was also reported to cause hindered bone formation.^[^ ^38^ ^]^ The alleviated inflammation phase and efficient degradability of MQ_2_T_2_ in bone tissues would probably benefit bone repair.

**Figure 5:**
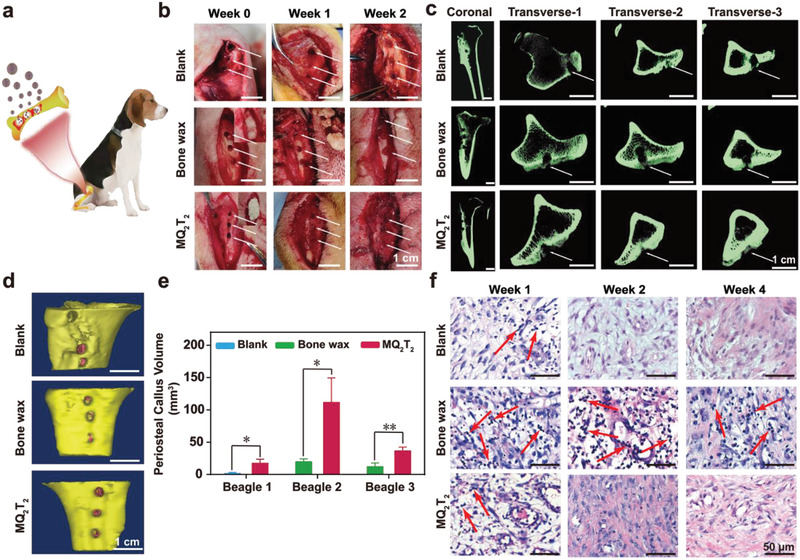
a) Proof‐of‐concept treatment with MQ_2_T_2_ in the cancellous bone defects of beagles. b) Representative photographs of bone apertures in the Blank, Bone wax and MQ_2_T_2_ groups during Week 0–2 (white arrow indicates bone apertures). c) Micro‐CT images of defected tibias samples, and d) 3D‐reconstructed photographs of typical bone apertures (yellow part indicates original bone and pink part indicates periosteal callus) in the Blank (Beagle 1), Bone wax (Beagle 3) and MQ_2_T_2_ (Beagle 3) groups at Week 4. e) Calculated volumes of periosteal callus in the Blank (Beagle 1), Bone wax (Beagle 2,3) and MQ_2_T_2_ (Beagle 1,2,3) groups from Week 1 to Week 4 (*n* = 3). f) Representative H&E staining analysis of muscles around defected tibias in the Blank, Bone wax and MQ_2_T_2_ groups during Week 1–4 (red arrow indicates inflammation‐associated cells). Data are presented as the mean ± SD. *p < 0.05, **p < 0.01 among the marked groups using multiple *t* tests.

In order to investigate the potential effect of MQ_2_T_2_ on large mammals, a beagle cancellous‐bone‐defect model was created as a proof‐of‐concept study (**Figure** **5**a). Figure 5b shows the representative photographs of bone apertures in three groups (Blank, Bone wax and MQ_2_T_2_) from Week 0 to Week 2. The bone apertures of the MQ_2_T_2_ group were observed to be covered with more and more connective tissues during healing process. On the contrary, the Blank and Bone wax groups still displayed naked bone apertures at Week 2. These results indicated that the MQ_2_T_2_ treatment contributed to the fastest repair of connective tissues on bone apertures at the early stage, which will be useful for controlling bone‐defect‐induced bleeding.

Tissue effusion of wounds was selected to evaluate the bleeding process of three groups. Obvious swelling of the wounds was observed in the Blank group (Beagle 1) at Week 1 and Week 2 (Figure S13, Supporting Information), which can be attributed to the serious chronic bleeding of cancellous bone defects and the subsequent tissue effusion generation. In the same Beagle 1, the MQ_2_T_2_ group exhibited a significantly lower amount of tissue effusion than the Blank group (0.1 g versus 1.4 g) at Week 2, indicating that MQ_2_T_2_ played a positive role in controlling the intractable bleeding induced by bone defects. In addition, no obvious swellings were observed in Beagles 2 and 3 for both MQ_2_T_2_ and Bone wax groups from Week 0 to Week 2. The results indicated that MQ_2_T_2_ group possessed the effective control of bone‐defect‐induced bleeding in beagles, which is comparable to commercial bone wax.

To evaluate bone repair process, the defected tibias of beagles were removed at Week 4 and assessed by micro‐CT (Figure 5c). For the Blank group (Beagle 1), two apertures at Transvese‐1 and Transvese‐3 sections were obviously observed and only the aperture at Transvese‐2 section was filled with some periosteal calluses. Compared with the Blank group, the MQ_2_T_2_ group in Beagle 1 exhibited a higher degree of bone aperture repair (Figure S10, Supporting Information). Particularly, the aperture at Transvese‐1 in the MQ_2_T_2_ group was totally filled with periosteal calluses. Moreover, the MQ_2_T_2_ group displayed a higher degree of bone aperture repair than the Bone wax group in Beagles 2 and 3 (Figure 5c; Figure S14, Supporting Information). The 3D‐reconstructed images of bone apertures and the quantitative data of periosteal calluses also confirmed that the MQ_2_T_2_ group produced the higher amount of periosteal calluses than the Bone wax and Blank groups (Figure 5d,e).

The muscles around the defected tibias were removed at Week 4 and performed with H&E staining analysis (Figure 5f). A large number of inflammation‐associated cells (indicated by red arrow) were generated in the Bone wax group from Week 1 to Week 4, revealing the poor biocompatibility of bone wax. On the contrary, due to the good biocompatibility of MQ_2_T_2_, the MQ_2_T_2_ group showed few inflammation‐associated cells at Week 1 and displayed a normal tissue morphology (well‐ordered normal cells, no inflammation‐associated cells) since Week 2. All the results of this proof‐of‐concept study confirmed the promising properties (hemostasis, bone repair, and biosafety) of MQ_2_T_2_ microparticles in cancellous‐bone‐defect models of large mammals, which will be of great potential in clinical utility.

## 3 Conclusions

In summary, multilayer‐structured MQ*_x_*T*_y_* microparticles exhibited efficient degradability, low cytotoxicity, and good blood compatibility. MQ_2_T_2_ with two Q^+^ layers and outmost T^−^ layer demonstrated the unique properties of platelet adhesion/activation and RBCs aggregation and produced the best in vitro hemostatic performance. In a mouse cancellous‐bone‐defect model, MQ_2_T_2_ displayed efficient control over intractable bleeding, low inflammation/immune responses and favorable degradation. In addition, MQ_2_T_2_ accelerated bone repair processes, which could further benefit the control of sustained bleeding. Finally, a proof‐of‐concept study of beagles further confirmed the promising properties of MQ_2_T_2_. Such biomass‐based absorbable microparticles, derived from plant polysaccharides and polyphenols, would provide a great potential for developing advanced biomedical materials to treat bone defects and/or intractable bleeding.

## 4 Experimental Section

##### Materials

Amylopectin from maize, tannic acid (T^−^), glucoamylase and *α*‐amylase were purchased from Sigma‐Aldrich (USA). Microporous starch particle from corn was purchased from Liaoning Li‐Da Bio‐Technique Co. Ltd (China). GTA and chloral hydrate were purchased from Energy Chemical (China). Bone wax was purchased from B.Braun Melsungen AG (Germany). Absorbable hemostat (Arista) was purchased from Medafor, Inc. (USA).

##### Preparation of Quaternized Starch (Q^+^)

Q^+^ was prepared by reacting amylopectin with GTA in the presence of NaOH. GTA (6 g) and NaOH (1.8 g) were dissolved into 40 mL and 10 mL of DI water, respectively. Then, GTA and NaOH solutions were mixed and the mixture was slowly dropped into a round‐bottom flask containing 6 g of amylopectin and 200 mL of DI water. Finally, the flask was kept stirring at room temperature for 24 h. The final reaction solution was dialyzed against DI water (MWCO, 1000 Da) for 2 days and lyophilized to produce Q^+^.

##### Preparation of Microporous Starch Particle with Q^+^ Layer (MQ)

As shown in Figure 1, Q^+^ was coated onto negatively charged microporous starch particle (M) via electrostatic assembly to prepare MQ. 0.6 g of Q^+^ was first dissolved into 30 mL of DI water in a round‐bottom flask. Then, 1.2 g of M was added into Q^+^ solution and the mixture solution was kept stirring at room temperature for 30 min. Finally, the produced MQ was purified from the mixture solution by centrifugation (10 000 rpm, 5 min), washing with DI water and lyophilization.

##### Preparation of Microporous Starch Particles with Multiple Q^+^/T^−^ Layers

1.2 g of T^−^ was first dissolved into 25 mL of DI water in a round‐bottom flask. Then, 1.0 g of MQ was added into T^−^ solution and the mixture solution was kept stirring at room temperature for 30 min. Finally, the produced microporous starch particle with double Q^+^/T^−^ layers (MQT) was purified from the mixture solution by centrifugation (10 000 rpm, 5 min), washing with DI water and lyophilization. For the preparation of microporous starch particle with triple Q^+^/T^−^ layers (MQ_2_T), 0.6 g of Q^+^ was first dissolved into 25 mL of DI water in a round‐bottom flask. Then, the prepared MQT (purified by centrifugation) was added into Q^+^ solution and the mixture solution was kept stirring at room temperature for 30 min. Finally, the produced MQ_2_T was purified from the mixture solution by centrifugation (10 000 rpm, 5 min), washing with DI water and lyophilization. These procedures were further repeated to produce microporous starch particle with quadruple and quintuple Q^+^/T^−^ layers (MQ_2_T_2_ and MQ_3_T_2_), with the fixed amount of T^−^ (1.2 g) or Q^+^ (0.6 g). Notably, for the preparation of microporous starch particles with multiple Q^+^/T^−^ layers, lyophilization was optional in the intermediate steps.

##### Physical Characterization

The chemical structure of Q^+^ was determined by nuclear magnetic resonance (NMR) spectroscopy (Bruker ARX 400 MHz, Germany). The morphologies of MQ*_x_*T*_y_* particles were observed by scanning electron microscope (SEM, Zeiss Supra 55, Germany). The particle size was calculated by analyzing SEM graph using Nano measure1.2. The *ζ* potential in water was measured by a Zetasizer (Malvern Nano ZS, UK). The chemical structure of MQ_2_T_2_ was determined by NMR solid spectroscopy (Bruker ARX 300 MHz, Germany). To investigate the solid‐like mechanical response, the rheological properties of MQ*_x_*T*_y_* particles were determined by Rheometer (View discorvery HR‐3, TA instruments, US), using similar procedures in previous paper.^[^ ^27^ ^]^ 400 µL of DI water and 0.2 g of samples were loaded onto the fixed lower plate (Diameter of 20 mm). Oscillation frequency measurements were designed in range from 0.5 to 100 rad s^−1^ at a constant magnitude of strain 0.01.

##### In Vitro Study of Disassembly and Degradation Behaviors

To investigate the disassembly process, the T^−^ release behaviors of MQ_2_T, MQ_2_T_2_ and MQ_3_T_2_ particles were first measured by an UV spectrophotometer (Shimadzu UV‐2600, Japan). After incubation with PBS at 37 °C for 6 h, disassembled MQ_2_T (or MQ_2_T_2_ or MQ_3_T_2_) was characterized by SEM observation and *ζ* potential measurement. Enzymolysis assay of MQ_2_T_2_ was performed using glucoamylase and amylase. The detailed process was available in the Supporting Information.

##### Cell Viability and Hemolysis Assay

The cell viabilities of MQ_2_T, MQ_2_T_2_ and MQ_3_T_2_ particles were evaluated by 3‐(4,5‐dimethyl‐2‐thiazolyl)‐2,5‐diphenyl‐2‐H‐tetrazolium bromide (MTT) assay with L929 cell line. Whole blood from healthy rabbit (female, 2–2.5 kg, obtained from Beijing Jinmunyang Experimental Animal Husbandry, China) was used in hemolysis assay. The detailed process was available in the Supporting Information.

##### In Vitro Hemostatic Assay

Fresh whole blood was drawn from Sprague‐Dawley (SD) rats (male, 200–250 g, obtained from Spaifu biotechnology co. LTD, China), immediately stored in an anticoagulation tube with sodium citrate and used for in vitro hemostatic study. The BCI assay was performed according to modified procedures in previous paper.^[^ ^28^, ^29^ ^]^ Platelet adhesion abilities of MQ_2_T, MQ_2_T_2_ and MQ_3_T_2_ were measured by a LDH assay.^[^ ^17^ ^]^ Platelet activation properties of MQ_2_T, MQ_2_T_2_, and MQ_3_T_2_ were measured by a P‐selectin (CD62P) assay.^[^ ^30^ ^]^ RBC adhesion properties of MQ_2_T, MQ_2_T_2_, and MQ_3_T_2_ were evaluated in diluted whole blood, using modified procedures.^[^ ^31^ ^]^ The detailed process was available in the Supporting Information.

##### In Vivo Study of MQ_2_T_2_ in a Mouse Cancellous‐Bone‐Defect Model

In vivo cancellous bone defect was first performed in SD rats (male, 200 g‐250 g) and approved by the Ethical Committee of the General Hospital of the People's Liberation Army (PLAGH), where chronic/osmotic cancellous bone bleeding was created. Briefly, SD rats were first anesthetized using 1 mL of 10% chloral hydrate solution. Then, the skin and muscle of right hind limb were incised on the medial part of tibia. Finally, a cylindrical bone aperture (Φ2*3 mm) was created from the medial side (through cortical bone to cancellous bone) using a round bur. A total of 61 SD rats (male, 200 g‐250 g) were randomly assigned to four groups: the Pristine (sacrified immediately after cancellous bone defect operation, assigned with 4 SD rats), Blank (cancellous bone defect without any treatment, assigned with 18 SD rats), Bone wax (cancellous bone defect filled with about 16 mg of bone wax into bone aperture, assigned with 21 SD rats) and MQ_2_T_2_ (cancellous bone defect filled with about 16 mg of MQ_2_T_2_ into bone aperture, assigned with 18 SD rats) groups. In addition, the skin and muscle were sutured after surgery and there was no additional wound around the bone aperture.

In order to evaluate the healing process, the SD rats in the Blank, Bone wax and MQ_2_T_2_ groups were sacrificed at the time points of 1, 2, 4, and 7 days after operations. The skin and muscle of right hind limb were cut open and the photographs of wound were captured. Some muscle parts around the bone aperture were soaked in polyformaldehyde solution (4%) and handled by a standard procedure for paraffin section and Congo red staining. 2 mL of the whole blood was extracted to perform blood biochemical assay (HGB, LY, and Eos). Moreover, the rest of whole blood was centrifuged at 2000 rpm for 20 min and the top plasma layer was collected for IL‐6 and TNF‐*α* analysis by ELISA kits (BlueGene Biotech., China). Finally, the right hind limb tibia was extracted from sacrificed rats (by euthanasia) and soaked in polyformaldehyde solution (4%). Bone samples were imaged by micro‐computed tomography (micro‐CT) (Gamma Medica‐Ideas, USA). The X‐ray was operated under the voltage of 75 kV and electricity of 135 µA. The scanning angle was 360° with a step of 0.40°. The imaging three‐dimensional data were reconstructed by minics 10.01. New bone and old bone were differentiated by bone density. After micro‐CT imaging, the bone samples were handled by a standard procedure for paraffin section, H&E staining and immunohistochemical staining.

##### A Prospective Study of MQ_2_T_2_ in a Beagle Cancellous‐Bone‐Defect Model

Beagle experiment (No. 2018‐D14‐21) was approved by the Ethical Committee of PLAGH and performed under legal protocol. Three beagles (male, ≈10 kg, named as Beagle 1, Beagle 2 and Beagle 3) were obtained from Beijing Ke‐Yu animal Breeding Center co. LTD (Beijing, China) and maintained under specific‐pathogen free condition. A cancellous bone defect model of beagle was performed to create cancellous bone bleeding in giant mammals, according to the similar procedures in SD rat model. Notably, the beagles were anesthetized using 13.5 mL of 3% pento‐barbital sodium solution. Both the right and left hind limb tibias of beagles were created with three cylindrical bone apertures (Φ3*5 mm). For the treatment, each three bone apertures on the left/right hind limb tibias of Beagle 1 (right), Beagle 2 (left), and Beagle 3 (right) were filled with about 500 mg of MQ_2_T_2_ and assigned to the MQ_2_T_2_ group. The three bone apertures on the left hind limb tibia of Beagle 1 received no treatment and was assigned to the Blank group. Both three bone apertures on the left/right hind limb tibias of Beagle 2 (right) and Beagle 3 (left) were filled with about 500 mg of bone wax and assigned to the Bone wax group. The skin and muscle were sutured after surgery and the beagle was observed walking normally during the period of healing.

In order to evaluate the healing process, the skin and muscle of the right and left hind limb tibias were cut open and the photographs of wound were captured at the time points of 1 and 2 weeks after operation. In addition, wound exudate (tissue effusion) of three groups was captured and collected using a syringe at time points of 1 and 2 weeks. After 4 weeks, the right and left hind limb tibias were removed and soaked in polyformaldehyde solution (4%) after the beagle was sacrificed using euthanasia. The obtained bone samples were imaged by micro‐CT) (Gamma Medica‐Ideas, USA), using the same parameters as mentioned above. Some muscle samples around the bone aperture (at time points of 1, 2, and 4 weeks) were soaked in polyformaldehyde solution (4%) and handled by a standard procedure for paraffin sections and H&E staining.

##### Statistical Analysis

All experiments were repeated at least three times(*n* ≥ 3). All measurement data followed a normal distribution, the results are expressed as mean ± standard deviation (SD). The differences between independent groups were tested by multiple *t* tests. In all pictures, p* < 0.05 represented one star, p** < 0.01 represented two stars, *p* ^***^ < 0.001 represented three stars, *p* ^****^ < 0.0001 represented four stars. Statistical analysis was carried out using GraphPad Prism 6 Software.

## Conflict of Interest

The authors declare no conflict of interest.

## Supporting information

Supporting Information
