# Supplementary material for: Biomass‐Derived Multilayer‐Structured Microparticles for Accelerated Hemostasis and Bone Repair
Source: Adv Sci (Weinh). 2020 Oct 4;7(22):2002243. doi: 10.1002/advs.202002243 (PMC7675182; doi:10.1002/advs.202002243)
Supplement: Supplementary file 1 — Supporting Information [file ADVS-7-2002243-s001.pdf]

© 2020 Wiley-VCH GmbH

ADVANCED SCIENCE Open Access

# Supporting Information

for *Adv. Sci.*, DOI: 10.1002/advs.202002243

# Biomass-Derived Multi-Layer-Structured Microparticles for Accelerated Hemostasis and Bone Repair

*Jia-Ying Liu, Yang Hu, Long Li, Chao Wang, Jia Wang, Yang Li,* Dafu Chen, Xiaokang Ding, Chuanan Shen,* and Fu-Jian Xu**

# Supporting Information

## Biomass-Derived Multi-Layer-Structured Microparticles for Accelerated Hemostasis and Bone Repair

*Jia-Ying Liu, Yang Hu, Long Li, Chao Wang, Jia Wang, Yang Li,* Dafu Chen, Xiaokang Ding, Chuanan Shen,* and Fu-Jian Xu**

J.-Y. Liu, Dr. Y. Hu, L. Long, C. Wang, J. Wang, Dr. Y. Li, Dr. X. Ding and Prof. F.-J. Xu
State Key Laboratory of Chemical Resource Engineering
Key Laboratory of Biomedical Materials of Natural Macromolecules (Beijing University of Chemical Technology, Ministry of Education)
Beijing Laboratory of Biomedical Materials
Beijing University of Chemical Technology
Beijing, 100029, China
E-mail: xufj@mail.buct.edu.cn (F.-J. Xu); ly@mail.buct.edu.cn (Y. Li)
Prof. D. Chen
Laboratory of Bone Tissue Engineering
Beijing Laboratory of Biomedical Materials
Beijing Research Institute of Traumatology and Orthopaedics
Beijing Jishuitan Hospital
Beijing, 100035, China
Prof. C. Shen
Department of Burn & Plastic Surgery
The First Affiliated Hospital of General Hospital of PLA
Beijing 100048, China
E-mail: shenchuanan@126.com (C. Shen)

## Materials and Methods

### *In vitro* study of disassembly and degradation behaviors

To investigate the disassembly process, the $T^-$ release behaviors of $MQ_2T$, $MQ_2T_2$ and $MQ_3T_2$ particles were firstly measured by an UV spectrophotometer (Shimadzu UV-2600, Japan). The UV absorbance (at 276 nm) of different concentrations of $T^-$ (1 μg/mL, 2 μg/mL, 4 μg/mL, 8 μg/mL, 16 μg/mL, 32 μg/mL, 64 μg/mL, 128 μg/mL) was tested to construct a standard curve. 4 mL of PBS (pH=7.4) and 2 mg of $MQ_2T$ (or $MQ_2T_2$ or $MQ_3T_2$) were incubated at 37 ℃ and the UV absorbance of the supernatant solution (at 276 nm, UV-Vis spectrometer) was measured at different time periods (0 h, 1/6 h, 1/3 h, 2/3 h, 5/6 h, 1 h, 2 h, 4 h, 6 h). Four duplicate samples were performed for each group. After incubation with PBS at 37 ℃ for 6 h, disassembled $MQ_2T$ (or $MQ_2T_2$ or $MQ_3T_2$) was separated by centrifugation (1000 rap/min, 1 min) and lyophilized, prior to SEM observation and ζ potential measurement. The precipitate was re-suspended with 1 mL water to measure ζ potential. Enzymolysis assay of $MQ_2T_2$ was performed using glucoamylase and amylase. 8 mg of glucoamylase and 2 mg of amylase were firstly dissolved in 20 mL of PBS (pH = 5.8) in a round-bottom flask and then 100 mg of $MQ_2T_2$ was added. The mixture was kept stirring at 37 ℃ and 1 mL of mixture was taken out at different time periods (1 h, 2 h, 3 h, 6 h, 16 h and 24 h). The degraded $MQ_2T_2$ was lyophilized, prior to SEM observation.

### Cell viability assay

The cell viabilities of $MQ_2T$, $MQ_2T_2$ and $MQ_3T_2$ particles were evaluated by 3-(4,5-dimethyl-2-thiazolyl)-2,5-diphenyl-2-H-tetrazolium bromide (MTT) assay with mouse fibroblast (L929) cell line, using modified procedures in previous paper.[S1] 160 mg of $MQ_2T$ (or $MQ_2T_2$ or $MQ_3T_2$) was added into 1 mL of typical culture medium

(Dulbecco's modified eagle medium (DMEM) containing 10 % heat-inactivated fetal bovine serum (FBS), 100 mg $mL^{-1}$ streptomycin and 100 mg $mL^{-1}$ penicillin, then incubated at 37℃ for 24 h, and finally filtered via sterile membranes (average pore size of 0.4 μm) to get stock leaching solution (160 mg/mL). 100 μL of stock leaching solution was diluted with 900 μL of typical culture medium (16 mg/mL) and further diluted with four concentration gradients (8, 4, 2 and 1 mg/mL). $2\times10^4$ of L929 cells in 100 μL of typical culture medium were seeded in each well of a 96-well cell plate and incubated at 37 ℃ for 24 h. Then, the culture media were replaced with as-prepared leaching solutions at five different concentrations and incubated for 24 h. The subsequent procedures of MTT staining and measure absorbance at 570 nm were the same with those described in our previous work.[S1] Five duplicate samples were performed for each group.

**Hemolysis assay**

Firstly, 2 mL of whole blood from healthy rabbit (female, 2-2.5 kg, obtained from Beijing Jinmunyang Experimental Animal Husbandry, China) was mixed with 20 mL of normal saline, centrifuged at 1500 rpm for 15 min to remove the supernatant liquid, and washed three times with saline. The collected precipitate was re-suspended in normal saline to prepare red blood cell (RBC) suspension (at the volume concentration of 2 %). RBC suspensions were incubated with $MQ_2T$ (or $MQ_2T_2$ or $MQ_3T_2$) at various concentrations (1, 2, 4, 8, 16 mg/mL), deionized (DI) water (positive control) and normal saline (negative control) for 3 h at 37 ℃. The hemolysis ratio was determined by the subsequent measurement of absorbance at 545 nm, using the same procedures with those described in our previous work.[S1] Four duplicate samples were performed for each group.

**In vitro hemostatic assay**

Fresh whole blood was drawn from Sprague-Dawley (SD) rats (male, 200 g-250 g, obtained from Spaifu biotechnology co. LTD, China), immediately stored in an anticoagulation tube with sodium citrate and used for *in vitro* hemostatic study. The blood-clotting index (BCI) assay was performed according to modified procedures in previous paper. [S2,S3] 10 μL of whole blood was dropped onto 5 mg of $MQ_2T$ (or $MQ_2T_2$ or $MQ_3T_2$ or medical gauze as the control group) in a plastic centrifuge tube. Each group was immediately added with $CaCl_2$ aqueous solution (1 μL, 0.2 M) and incubated at 37 ◦C for 5 min. Thereafter, 2 mL of DI water was carefully added into the centrifuge tube to break/dissolve the unclotted RBCs, which was further incubated at 37 ◦C for 5 min. Finally, the different suspensions were centrifuged (500 rpm, 1 min) and the supernatant liquid was collected. The hemoglobin (HGB) content of each group was determined by the subsequent measurement of absorbance at 520 nm ($Abs_{sample}$), using the same procedures with those described in our previous work (26). The HGB content of citrated whole blood (10 μL, in 2 mL DI water) was measured as the reference ($Abs_{Blank}$). Three duplicate samples were performed for each group. The blood-clotting index (BCI) of $MQ_xT_y$ in recalcified whole blood was calculated from the following equation: $BCI_{(with\ CaCl2)}$ index = $Abs_{sample}$ /$Abs_{blank}$ × 100 %. Meanwhile, the $BCI_{(without\ CaCl2)}$ index of $MQ_x$Ty in citrated whole blood was also calculated, using the same procedures without addition of $CaCl_2$ aqueous solution.

Platelet adhesion abilities of $MQ_2T$, $MQ_2T_2$ and $MQ_3T_2$ were measured by a lactate dehydrogenase (LDH) assay, according to previous paper.[S4] Fresh citrated whole blood of SD rat was centrifuged at 1500 rpm for 15 min to obtain platelet rich plasma (PRP). PRP was diluted with PBS to be $2*10^8$/mL. 5 mg of $MQ_2T$ (or $MQ_2T_2$ or $MQ_3T_2$ or gauze as control) was incubated with 100 μL of diluted PRP suspension in a plastic centrifuge tube at 37 ℃ for 30 min. Then, the supernatant liquid was discarded

and the non-adhesion platelets were removed by PBS washing. The sample was transferred to a new centrifuge tube and incubated with 250 μL of Triton X-100 lysate (1 %, DI water) at 37 ℃ for 1 h, to lyse the adherent platelets. Finally, the lysates were measured by the LDH assay Kit (Shanghai Dongren chemical technology, Co., Ltd, China) to quantify the number of adherent platelets. Diluted PRP suspensions at various concentrations (0.5, 1.5, 2.0, 3.0, 4.0 $*10^6$/mL) were also lysed and measured by the LDH assay Kit to obtain a standard curve. Platelet activation properties of $MQ_2T$, $MQ_2T_2$ and $MQ_3T_2$ were measured by a P-selectin (CD62P) assay, according to previous paper.[S5] PRP of SD rat was obtained from fresh citrated whole blood as mentioned above. 10 mg of $MQ_2T$ (or $MQ_2T_2$ or $MQ_3T_2$ or gauze as control) was added with 200 μL of PRP in a plastic centrifuge tube and incubated at 37 ◦C for 3 min. Thereafter, the supernatants were collected from different suspensions and measured by the Rat P-selectin Elisa Kit (Shanghai Xinfan Biotechnology, Co., Ltd, China) to quantify the amount of CD62P.

RBC adhesion properties of $MQ_2T$, $MQ_2T_2$ and $MQ_3T_2$ were evaluated in diluted whole blood, using modified procedures.[S6] Fresh citrated whole blood of SD rat was firstly diluted with PBS (v/v, 1:19). 10 mg of $MQ_2T$ (or $MQ_2T_2$ or $MQ_3T_2$ or gauze as control) was incubated with 400 μL of diluted PRP suspension in a plastic centrifuge tube. The suspensions were vortexed for 2 min and then incubated at 37 ℃ for 60 min. The supernatants were collected from different suspensions and the non-adhesion RBCs were removed by PBS washing (200 μL). Such two supernatants were mixed and measured with the absorbance at 520 nm to determine the amount of RBC (HGB content). Meanwhile, the samples with adhered RBC were immobilized with aqueous glutaraldehyde solution (2.5%, for 2h), dehydrated with ethanol (60%, 70%, 80%, 90%, and 100%, each for 10 min) and dried at air, prior to SEM observation.

*Statistical analysis:*

All experiments were repeated at least three times($n \geqslant 3$). All measurement data followed a normal distribution, the results are expressed as mean ± standard deviation (SD). The differences between independent groups were tested by multiple t tests. In all pictures, $P^{*} < 0.05$ represented one star, $P^{**} < 0.01$ represented two stars, $P^{***} < 0.001$ represented three stars, $P^{****} < 0.0001$ represented four stars. Statistical analysis was carried out using GraphPad Prism 6 Software.

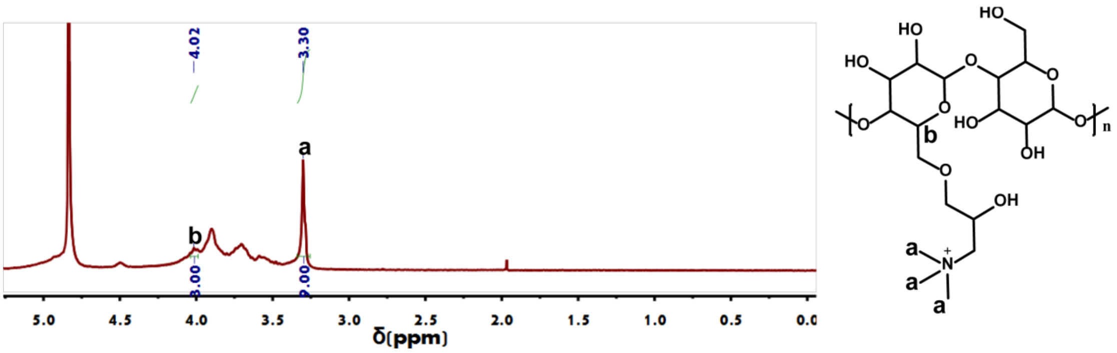


**Figure S1** $^1$H NMR spectrum of $Q^+$

**Analysis.** For $Q^+$ (Figure S1), the chemical shift at about 3.30 ppm is attributed to (a) $N^+$-$\underline{CH_3}$ methyl, while the chemical shift at about 4.02 ppm is attributed to the (b) $\underline{CH}$-$CH_2$-O methine proton on glucose units of amylopectin. Based on the area ratio of peak a and peak b, the average substitution degree of quaternary ammonium was calculated to be about 33 % (of glucose units) in cationic starch.

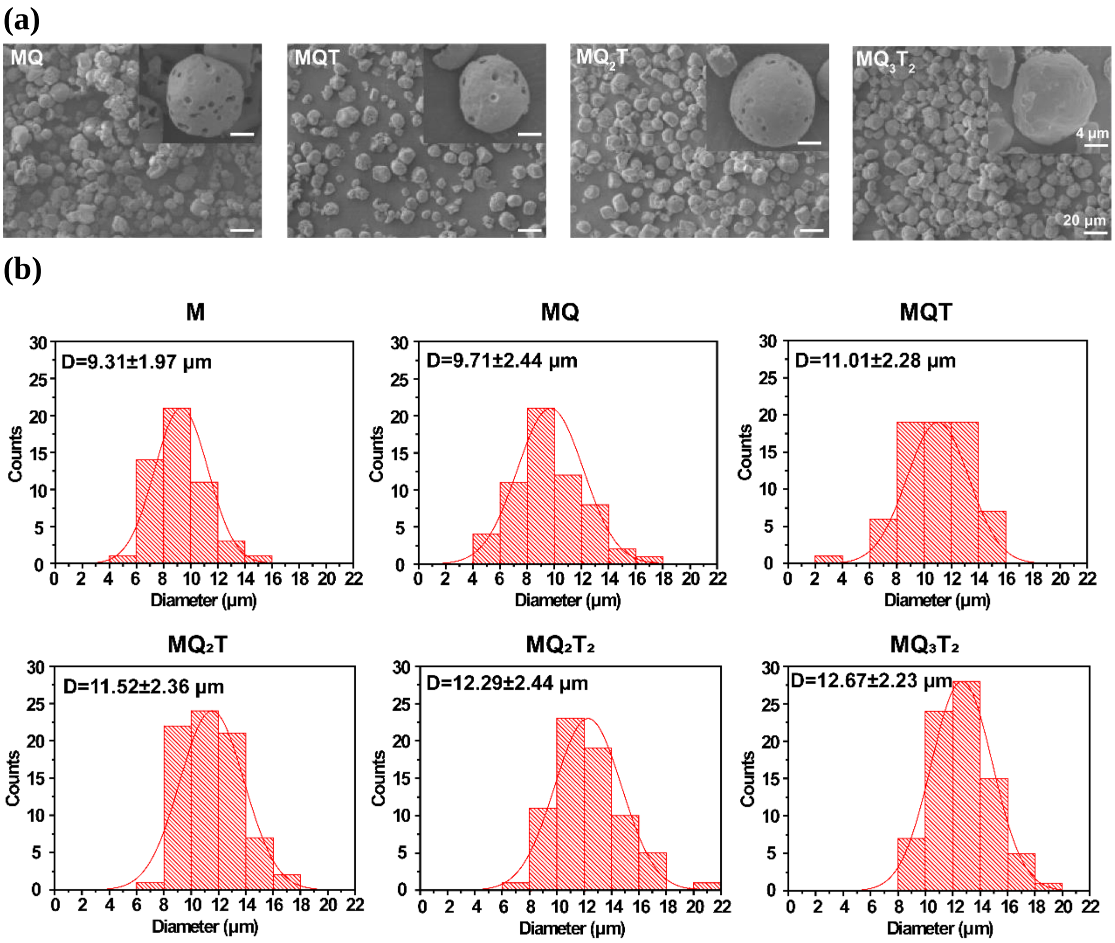


**Figure S2**. (a) SEM images of $MQ_xT_y$ particles (magnified images in the inserts). (b) The statistical data about the particle size of M and $MQ_xT_y$ microparticles in water (before incubation with PBS).

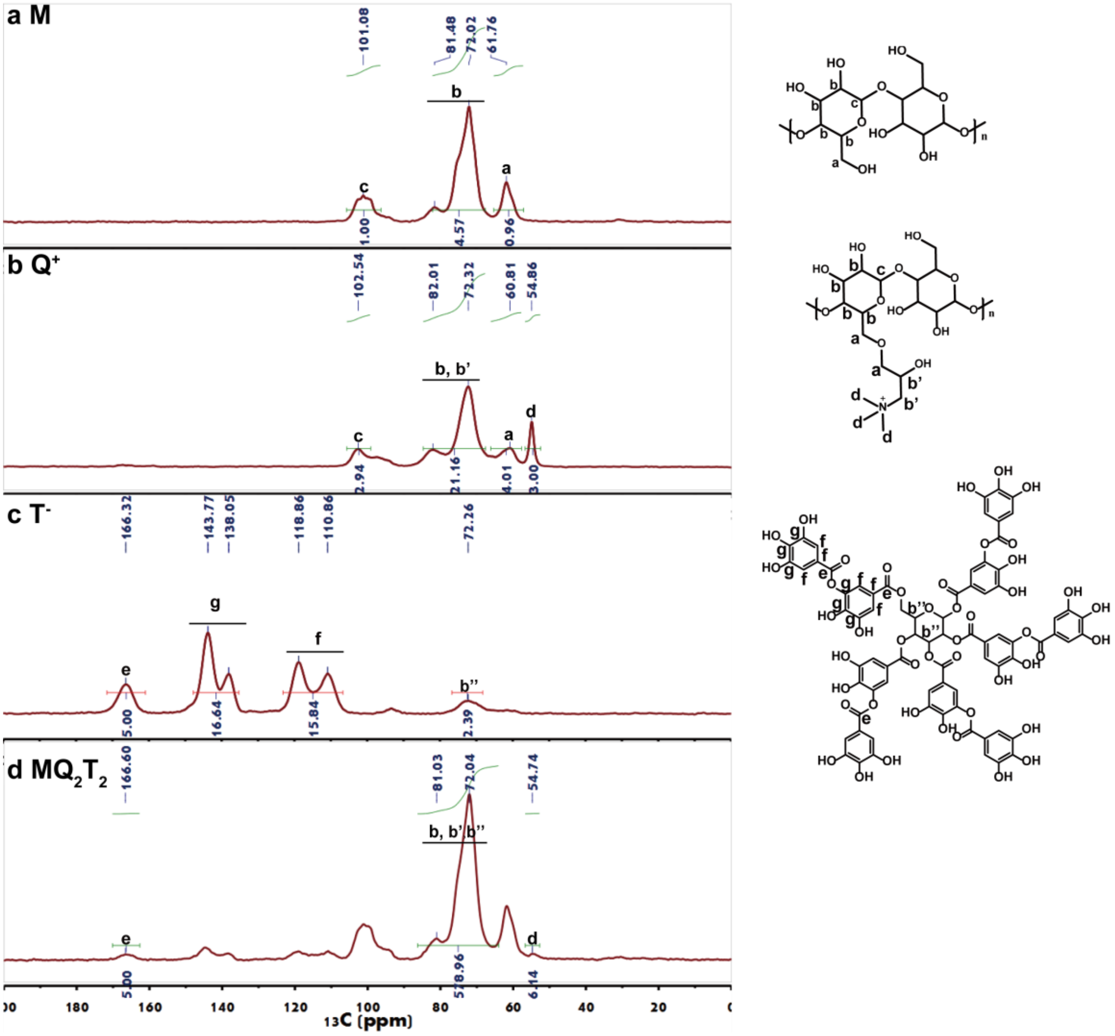


**Figure S3.** $^{13}$C NMR spectra of M, $Q^+$, $T^-$ and $MQ_2T_2$.

**Analysis.** For M (Figure S2a), the chemical shifts at 61.76 and 101.08 ppm are attributable to (a) $CH_2$-OH methylene and (c) O-CH-O methine on glucose units of microporous starch, respectively. The chemical shifts at 72.02 ppm-81.48 ppm are attributable to the inner methine (b, CH-O) on glucose units of M.

For $Q^+$ (Figure S2b), the chemical shifts at 60.81 and 102.54 ppm are attributable to (a) $CH_2$-O-$CH_2$ methylene and (c) O-CH-O methine on glucose units of microporous starch, respectively. The chemical shifts at 72.32 ppm-82.01 ppm are attributable to the inner methine (b, CH-O) on glucose units and the methine/methylene (b', HO-CH and $N^+$-$CH_2$) of the GTA substituent groups. The chemical shift at 54.86 ppm is attributable to the carbon atom of the methyl (d) $N^+$-$CH_3$ of the GTA substituent groups.

For $T^-$ (Figure S2c), the chemical shifts at 72.26 and 166.32 ppm are attributable

to the carbon atoms of the (b’’) O-CH-CH methine and (e) O=C-O of $T^-$, respectively. The chemical shifts at 110.86 ppm-118.86 ppm and 138.05 ppm-148.77 ppm are attributable to the carbon atoms of (f) C-CH and (g) C-OH on benzene ring.

For $MQ_2T_2$ (Figure S2d), the existences of peak b-b’’, peak d and peak e and their area ratios confirm that $MQ_2T_2$ was composed of M, $Q^+$ and $T^-$.

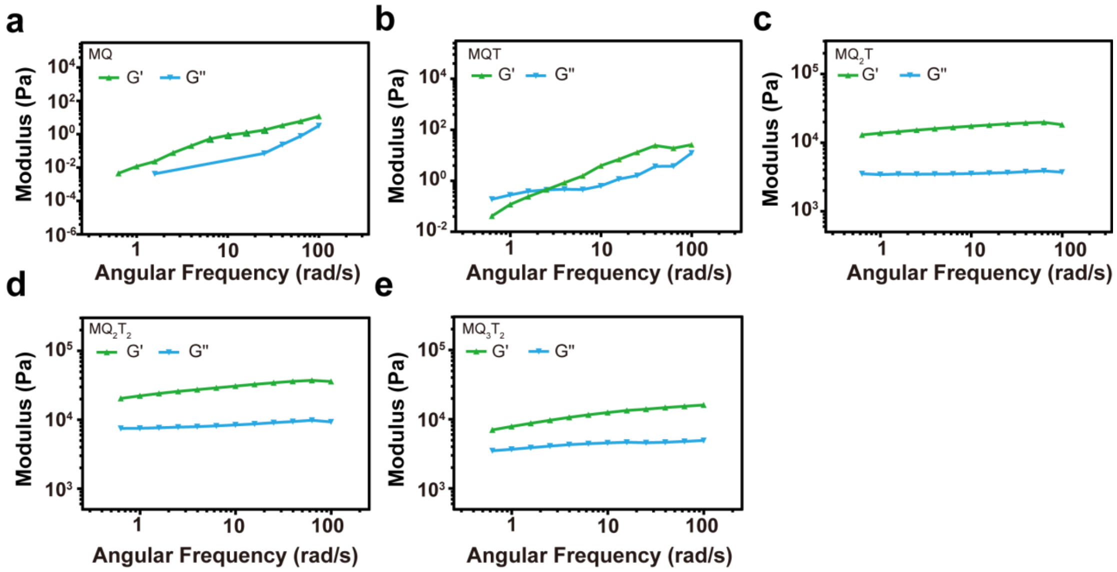


**Figure S4.** Oscillation frequency sweep curves of $MQ_xT_y$ particles.

**(a)**

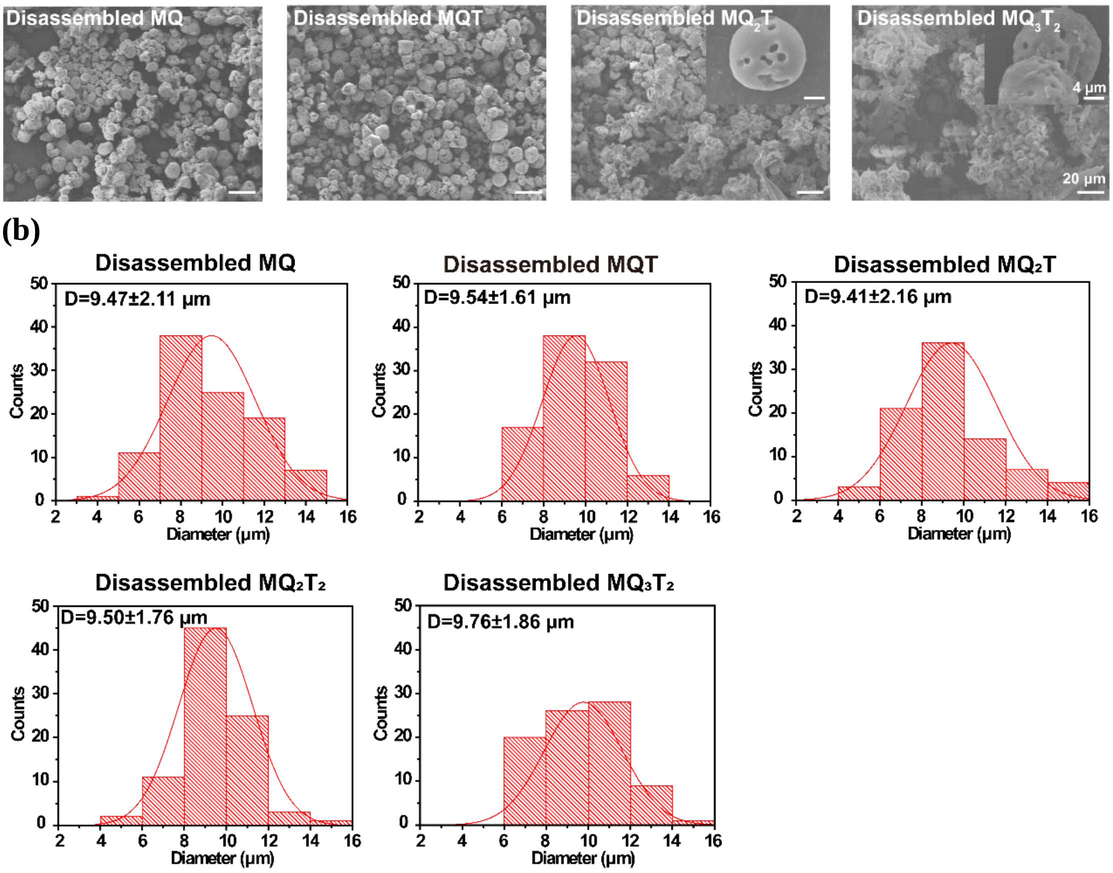


**Figure S5.** (a) SEM images of disassembled MQ, MQT, $MQ_2T$ and $MQ_3T_2$ particles incubated with PBS at 37 ℃ for 6 h (magnified images of disassembled $MQ_2T$ and $MQ_3T_2$ in the inserts). (b) The statistical data about the particle size of MQxTy microparticles after incubation with PBS.

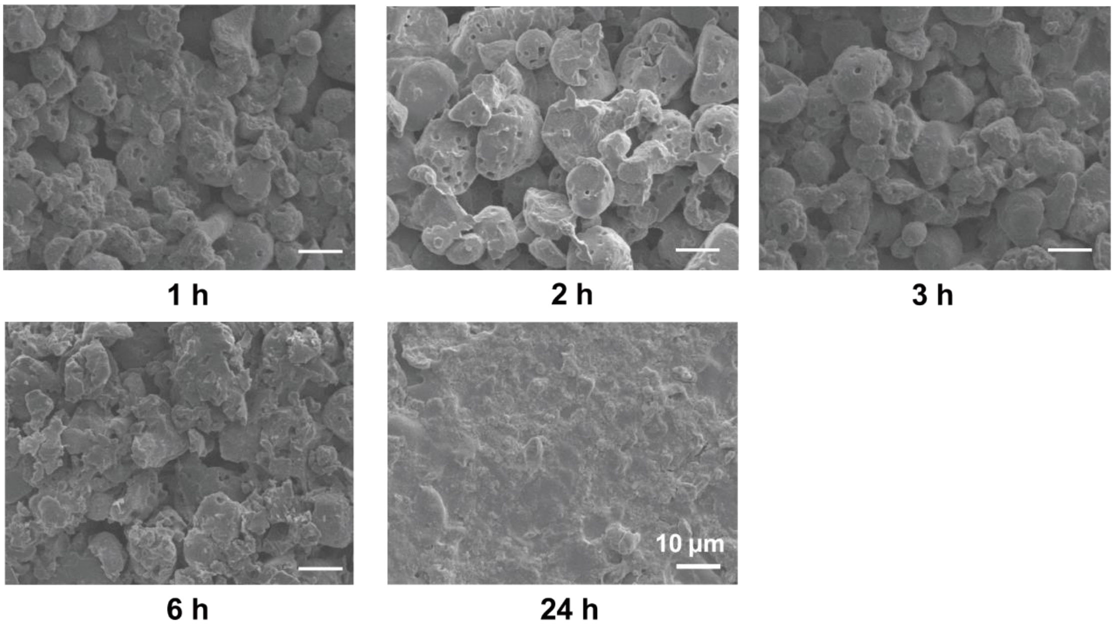


**Figure S6.** SEM images of degraded $MQ_2T_2$ after the treatment with glucoamylase/amylase for 1 h, 2 h, 3 h, 6 h and 24 h.

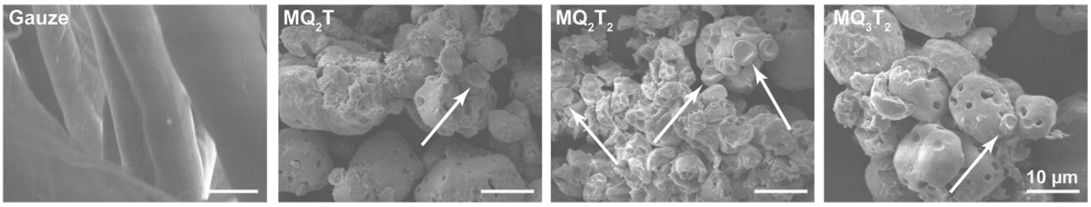


**Figure S7.** SEM images of adherent RBCs in gauze, $MQ_2T$, $MQ_2T_2$ and $MQ_3T_2$ after incubation with diluted whole blood (white arrow indicates RBCs).

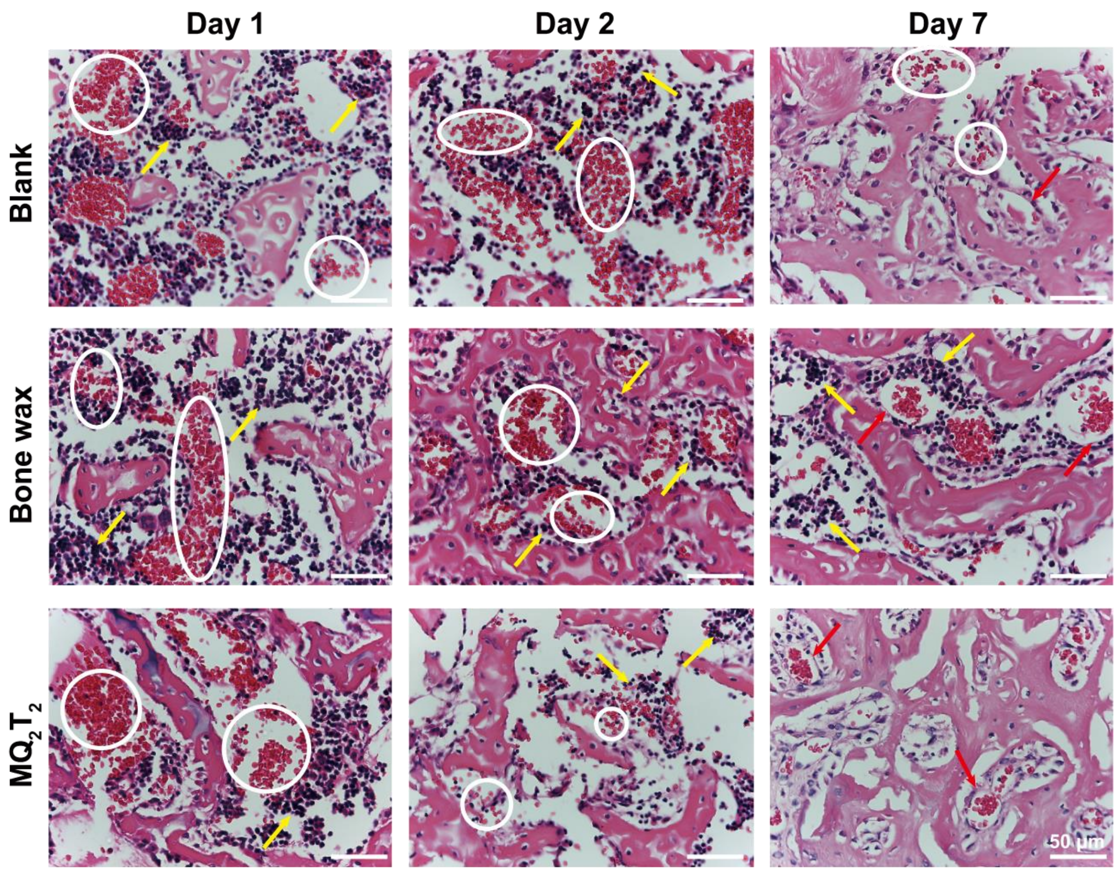


**Figure S8.** H&E staining analysis of defected bone samples in the Blank, Bone wax and $MQ_2T_2$ groups at Day 1, Day 2 and Day 7 (white circle indicates bleeding sites, yellow arrow indicates inflammatory cells and red arrow indicates repaired blood vessels).

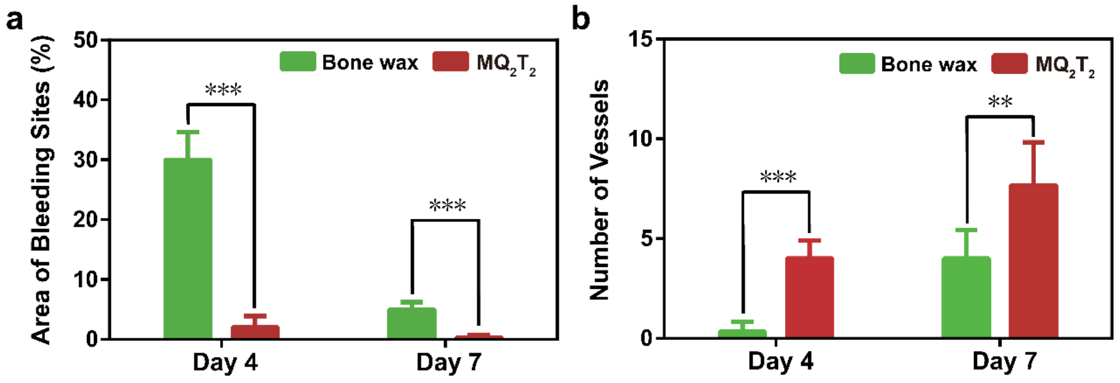


**Figure S9.** (a) Bleeding sites area and (b) number of repaired/complete blood vessels (area: 220 μm*300 μm) of defected bone samples in the Bone wax and $MQ_2Q_2$ groups at Day 4 and Day 7(n=6). Data are presented as the mean ± SD.*$p < 0.05$, **$p < 0.01$, ***$p < 0.001$ among the marked groups using multiple t tests.

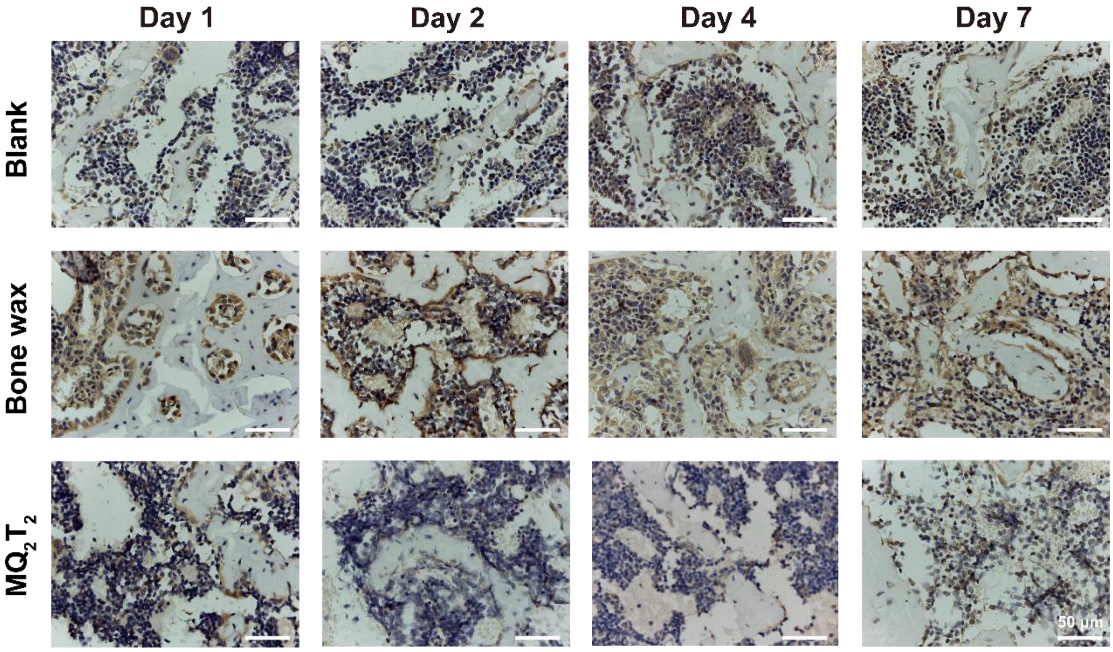


**Figure S10.** Immunohistochemical staining analysis of defected bone samples in the Blank, Bone wax and $MQ_2T_2$ groups from Day 1 to Day 7, where brown part indicates (a) TNF-α and (b) IL-6expression.

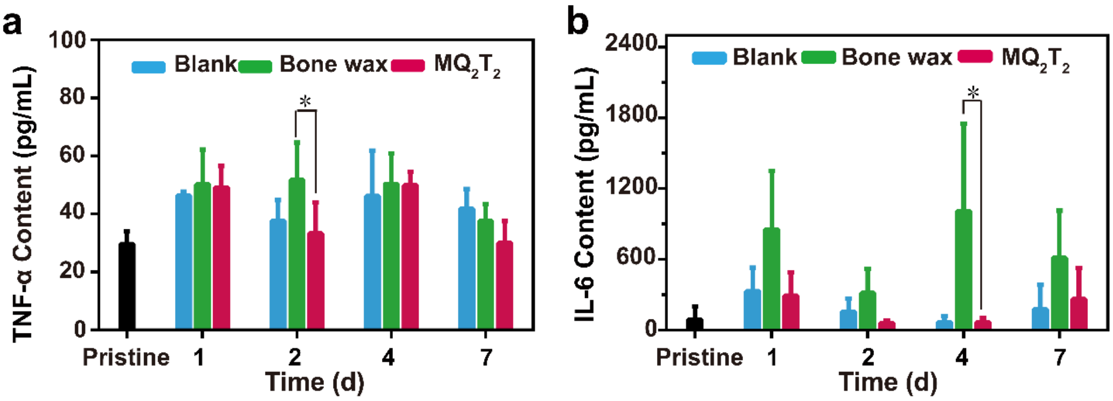


**Figure S11.** (a) TNF-α and (b) IL-6 contents of whole blood samples in the Blank, Bone wax and $MQ_2T_2$ groups from Day 1 to Day 7 (n=3-7). Data are presented as the mean ± SD.*$p < 0.05$ among the marked groups using multiple t tests.

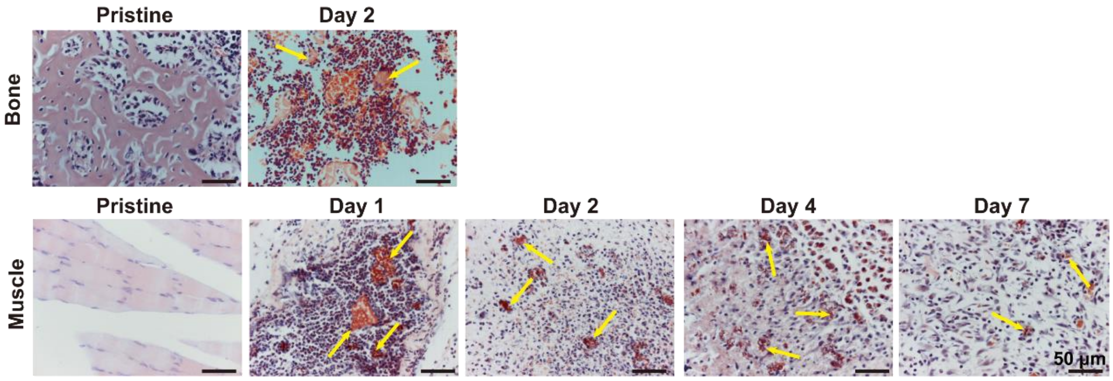


**Figure S12.** Congo red staining analysis of bone (Day 0 and Day2) and surrounding muscle (Day 0 to Day 7) samples in the $MQ_2T_2$ group, where yellow arrow indicates Congo red-stained $MQ_2T_2$.

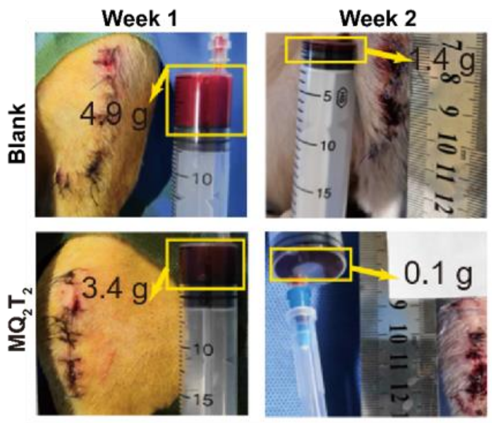


**Figure S13.** Photographs of tissue effusion of the wounds of Beagle 1 in the Blank and $MQ_2T_2$ groups at Week 1 and Week 2, where inserted numeral indicates the weights of tissue effusion.

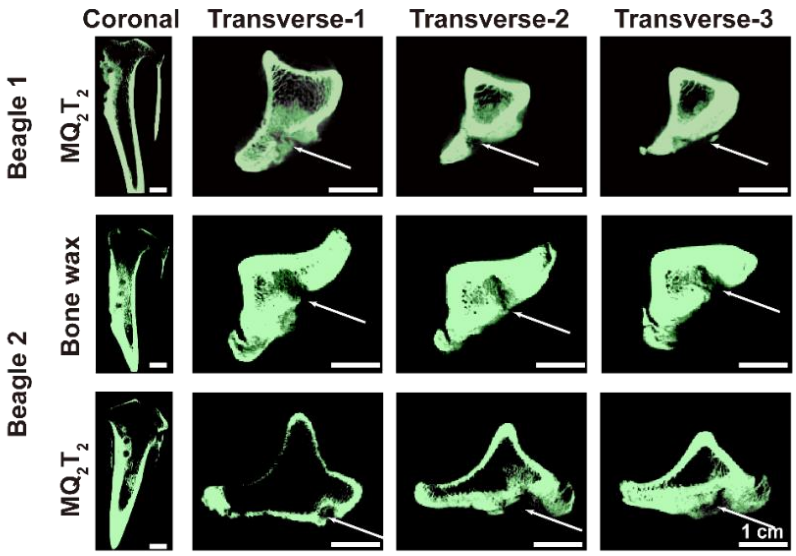


**Figure S14.** Micro-CT images of defected tibia samples in the Bone wax (Beagle 2) and $MQ_2T_2$ (Beagles 1,2) groups at Week 4.

## References

S1. Y. Hu, Z. Zhang, Y. Li, X. Ding, D. Li, C. Shen, F. J. Xu. *Macromol. Rapid Commun.* **2018**, *39*, 1800069.
S2. G. Lan, B. Lu, T. Wang, L. Wang, J. Chen, K. Yu, J. Liu, F. Dai, D. Wu. *Colloids Surf. B* **2015**, *136*, 1026.
S3. J. Liu, Y. Li, Y. Hu, G. Cheng, E. Ye, C. Shen, F. J. Xu. *Mater. Sci. Eng. C* **2018**, *83*, 160.
S4. F. Chen, X. Cao, X. Chen, J. Wei, C. Liu. *J. Mater. Chem. B* **2015**, *3*, 4017.
S5 X. Shi, Q. Fang, M. Ding, J. Wu, F. Ye, Z. Lv, J. Jin. *J. Biomater. Appl.* **2016**, *30*, 1092.
S6. K. Quan, G. Li, L. Tao, Q. Xie, Q. Yuan, X. Wang. *ACS Appl. Mater. Interfaces* **2016**, *8*, 7666.
